# Nitrate and ammonium fluxes to diatoms and dinoflagellates at a single cell level in mixed field communities in the sea

**DOI:** 10.1038/s41598-018-38059-4

**Published:** 2019-02-05

**Authors:** Malin Olofsson, Elizabeth K. Robertson, Lars Edler, Lars Arneborg, Martin J. Whitehouse, Helle Ploug

**Affiliations:** 1 Department of Marine Sciences, University of Gothenburg, SE 405 30 Gothenburg, Sweden; 2 WEAQ AB, Doktorsgatan 9D, Ängelholm, Sweden; 3 Research and Development Department, Swedish Meteorological and Hydrological Institute, Norrköping, Sweden; 4 Swedish Museum of Natural History, Stockholm, Sweden

## Abstract

Growth of large phytoplankton is considered to be diffusion limited at low nutrient concentrations, yet their constraints and contributions to carbon (C) and nitrogen fluxes in field plankton communities are poorly quantified under this condition. Using secondary ion mass spectrometry (SIMS), we quantified cell-specific assimilation rates of C, nitrate, and ammonium in summer communities of large phytoplankton when dissolved inorganic nitrogen concentrations are low in temperate coastal regions. Chain-forming diatoms composed 6% of total particulate organic carbon, but contributed 20% of C assimilation, 54% of nitrate assimilation and 32% of ammonium assimilation within the plankton community. In contrast, large dinoflagellates composed 11% of total POC, and contributed 14% of the C assimilation, 4% of ammonium and 9% of nitrate assimilation within the plankton community. Measured cell-specific C and nitrate assimilation rate match the Redfield ratio and the maximal nitrate assimilation in *Chaetoceros* spp. predicted by mass transfer theory. However, average ammonium assimilation rates were 30 and 340% higher than predicted by mass transfer theory in *Tripos/Ceratium* and *Chaetoceros*, respectively, suggesting that microbial interactions in the phycosphere may facilitate substantial luxury ammonium uptake by *Chaetoceros* in environments with fluctuating nitrate concentrations.

## Introduction

Aquatic primary production constitutes approximately 50% of global primary production^1^. Nitrogen (N) globally limits the marine primary productivity, and bioavailable sources are mainly nitrate and ammonium^2^. In the ocean, biological CO_2_ sequestration from the atmosphere to the ocean interior is described by the primary production driven by new external N sources into the euphotic zone, e.g., N_2_ fixation or nitrate derived from land, or transports across the thermocline from deeper waters via upwelling or diapycnal mixing. Over longer time scales, this new production reflects the drawdown and transport of atmospheric CO_2_ to the ocean interior, i.e., the biological carbon pump^3,4^. Large, chain-forming diatoms form the base of the food web and play a major role in new production and the biological carbon pump by their CO_2_ assimilation and transport to the ocean interior as fast-sinking aggregates in, e.g., upwelling regions and during spring blooms where nitrate concentrations are high^5–7^. Thus, nitrate-rich ecosystems dominated by chain-forming diatoms are associated with a large proportion of new production. In contrast, ecosystems and communities with low dissolved inorganic nitrogen (DIN) concentrations where primary production is mainly based on small phytoplankton, are often associated with regenerated production (and N_2_ fixation)^8,9^. The oligotrophic (sub)tropical ocean and summer blooms in temperate coastal regions are characterized by low DIN concentrations and low abundances of large, chain-forming diatoms, while large, supposedly mixotrophic dinoflagellates, and picoplankton (<2 μm) often dominate the phytoplankton community. These observations have generally been explained by diffusion-limited growth of large phytoplankton cells, including chain-forming diatoms and dinoflagellates, at low DIN concentrations as demonstrated in monocultures in the laboratory^10–12^.

Mixing of water is inefficient at a micrometer scale where viscous forces dominate inertial forces. Transport of dissolved gases, nutrients, and dissolved organic matter (DOM) to and from phytoplankton cells and microorganisms therefore occurs by diffusion. Concentration gradients of gases, nutrients, and DOM consequently develop in the cell–water interface, e.g., active nutrient uptake across the cell membrane keeps the nutrient concentration lower at the cell surface relative to that in the ambient water^7,10,12^. The radius of this interface between the cell surface and the ambient water characterized by concentration gradients of gases, nutrients, and DOM can be 10 to 100 times larger than the cell and is referred to as the phycosphere^13^. Nutrient gradients at a micrometer scale are further enhanced by patchiness of released ammonium from zooplankton and protists^14^. Thus, nutrients are not evenly distributed on a microscale in the sea. Diffusion limited primary production occurs when the steady-state concentration of nutrients at the cell surface approaches zero and the maximum possible concentration gradient across the cell–water interface therefore limits uptake of a nutrient from the ambient water by the cell. Diffusion limited primary production occurs more likely in large cells or colonies of cells with higher nutrient demands compared to those in small, solitary cells when nutrient concentrations are low in the ambient water.

Secondary ion mass spectrometry (SIMS), a novel culture-independent technique in biological oceanography, is a high spatial resolution technique that combines the qualities of a microscope with those of a mass spectrometer, and measures isotopic composition at a resolution of ca. 1 μm. Combining this technique with stable isotopic tracer incubations in mixed field communities has revealed that growth rates in large diatom chains can be surprisingly high despite diffusion-limited nutrient supply when DIN concentrations are <0.3 μM in the sea^7,15^. Furthermore, it was demonstrated that carbon (C)-assimilation at a single cell level in chain-forming diatoms can be enhanced between 10% and 60% by turbulent strain relative to stagnant conditions in the euphotic zone^7^. With this technique, we can measure single-cell uptake within mixed field communities with their naturally low nutrient concentrations and organism interactions within the phycosphere intact^7,15–17^. In the present study, we hereby quantified assimilation of nitrate and ammonium into chain-forming diatoms and large dinoflagellates at a single-cell level within field populations and compared the empirical rates with those predicted by mass transfer theory. Thereby we were able to quantify diffusion limited growth in large cells at low DIN concentrations and even quantify their respective contributions to total C and N fluxes within the plankton community at low DIN concentrations during late summer in a temperate coastal sea.

## Results

### Community composition and nutrient concentrations

We performed our study in a plankton community in a temperate coastal region with persistently low nutrient concentrations during late summer when turbulence was moderate. We collected water at 5 m depth where the energy dissipation rate was 10^−6^ W kg^−1^ equal to a shear rate of 1 s^−1^ (Supplementary Fig. S1). Species diversity was high with more than 83 and 117 identified taxa of plankton >3 μm in August and September, respectively (Supplementary Table S1). By C-biomass, the community of planktonic organisms >3 μm consisted of 13% diatoms and 77% dinoflagellates in August, and of 30% diatoms and 64% dinoflagellates in September (Supplementary Table S2). Species belonging to *Tripos/Ceratium* spp., *Asterionellopsis glacialis*, *Chaetoceros* spp. (>8 µm) and other chain-forming diatoms ca. 5–20 µm (e.g., *Skeletonema marinoi*, *Cerataulina pelagica*, *Dactyliosolen* spp., *Leptocylindrus danicus*, *Guinardia delicatula*) were analyzed by SIMS to assess their assimilation of C, ammonium, and nitrate. Ambient concentrations of nitrate and ammonium varied between 0.17 and 0.59 μM, while orthophosphate and silicate concentrations were <0.3 μM and 5.7 μM, respectively (Supplementary Table S3).

### Cell-specific assimilation rates in dinoflagellates and chain-forming diatoms

**Figure 1:**
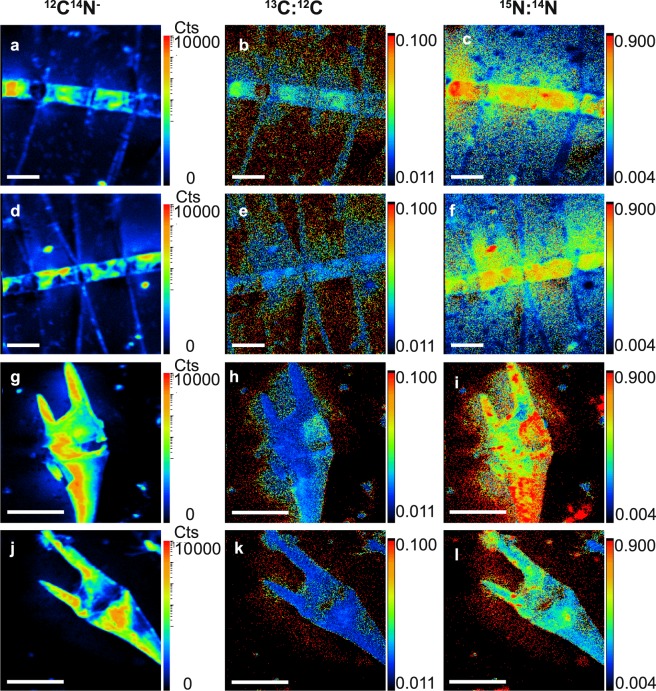
SIMS images of stable isotopes measured within phytoplankton. ^12^C^14^N^−^ (counts per pixel) (**a**,**d**,**g**,**j**), ^13^C:^12^C (dimensionless) (**b**,**e**,**h**,**k**), and ^15^N:^14^N (dimensionless) (**c**,**f**,**i**,**l**) measured within *Chaetoceros* spp. >8 µm; scale bar 20 μm (**a**–**f**) and *Tripos/Ceratium* spp. scale bar 50 μm (**g**–**l**). Cells were incubated with ^13^C bicarbonate and ^15^N nitrate (**a**–**c**,**g**–**i**) or ^13^C-bicarbonate and ^15^N ammonium (**d**–**f**,**j**–**l**).

**Figure 2:**
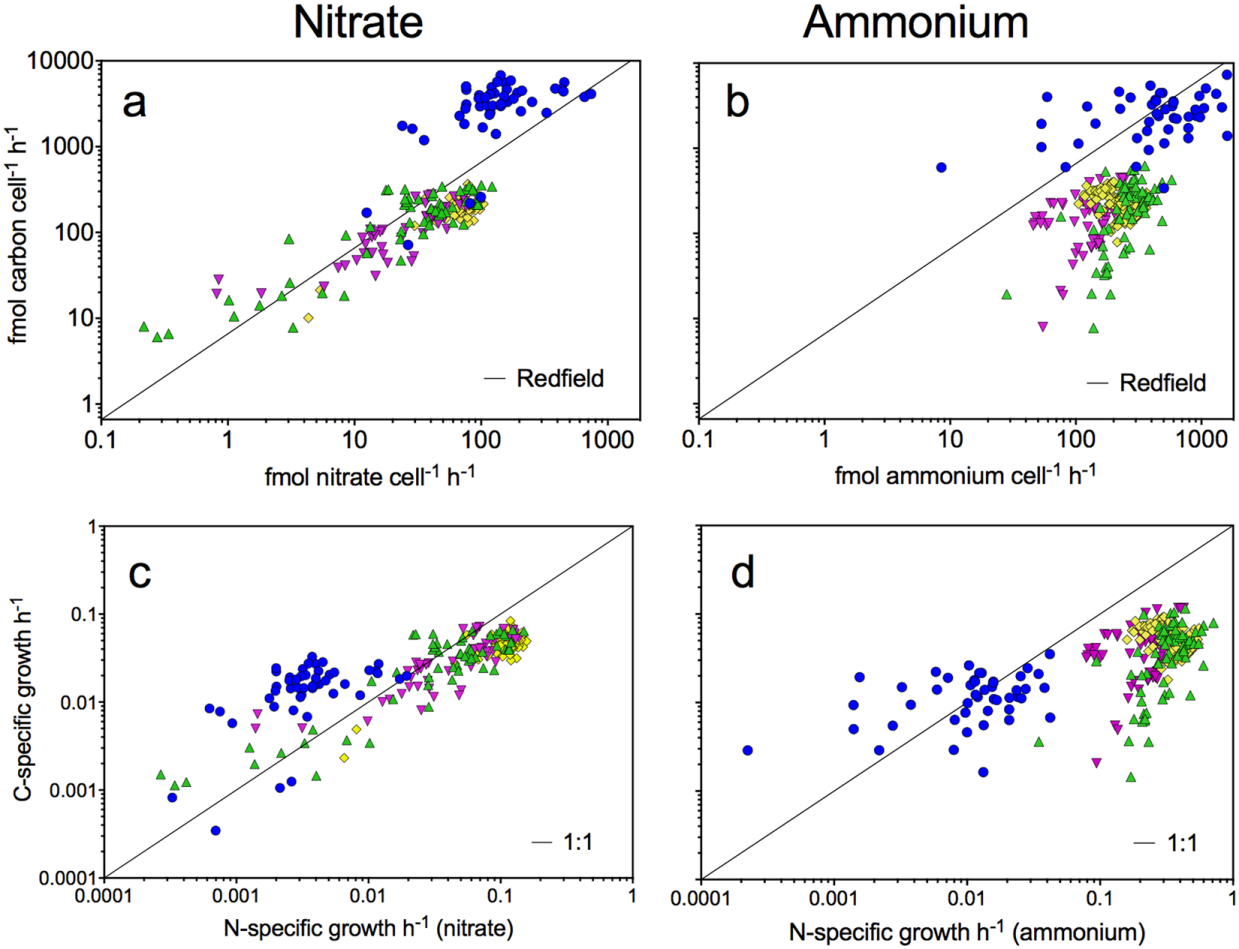
C and N assimilation at a single-cell level. The single-cell nitrate and ammonium assimilation rates (fmol cell^−1^ h^−1^) and single-cell carbon (C)- and nitrogen (N)-specific growth rates (h^−1^) for *Tripos*/*Ceratium* spp. (blue circles), *Chaetoceros* spp. >8 µm (green triangles), *Asterionellopsis glacialis* (yellow diamonds), and other chain-forming diatoms 5–20 µm (purple triangles). The black line indicates either Redfield (**a**,**b**) or a 1:1 ratio (**c**,**d**).

Examples of SIMS images of ^12^C^14^N^−^, ^13^C:^12^C and ^15^N:^14^N isotope ratios measured within cells of *Chaetoceros* spp. and *Tripos*/*Ceratium* spp. are shown (Fig. 1). The cell-specific C, nitrate, and ammonium assimilation rates (fmol cell^−1^ h^−1^) as well as C and N growth rates (h^−1^) shown in Fig. 2 were derived from the isotope ratios mapped within various chain-forming diatoms including *Chaetoceros* spp., and in large dinoflagellates, *Tripos*/*Ceratium* spp. (Eqs. 1–6).

The C and N growth rates (h^−1^) express the relative increase of C or N within cells independent of cell size and are therefore useful to compare growth rates among phytoplankton genera. C growth rates in the large *Tripos*/*Ceratium* spp. were on average 0.015 h^−1^ corresponding to a C-doubling time of approximately 3 days, while that of *Chaetoceros* spp. was ca. 1 d, only (Eq. 1; Table 1). Average N growth rate based on total DIN assimilation in chain-forming diatoms was more than an order of magnitude higher than that in *Tripos*/*Ceratium* spp., and could support doubling times of ca. 0.5 d (Eq. 2; Fig. 2 and Table 1). Despite the slower growth rates, the cell-specific C and N assimilation rates in *Tripos*/*Ceratium* spp. were substantially higher than those in the faster-growing diatoms because of their large cell size (Fig. 2).

**Table 1:** Taxa-specific assimilation rates based on single-cell measurements.

	N-specific ammonium assimilation h^−1^	Cell-specific ammonium assimilation fmol cell^−1^ h^−1^	Carbon to ammonium assimilation ratio	N-specific nitrate assimilation h^−1^	Cell-specific nitrate assimilation fmol cell^−1^ h^−1^	Carbon to nitrate assimilation ratio	C-specific carbon assimilation h^−1^	Cell-specific carbon assimilation fmol cell^−1^ h^−1^
**Diatoms**								
*Chaetoceros* spp. >8 µm	0.335 ± 0.015	271.9 ± 11.8	0.84 ± 0.06	0.051 ± 0.005	41.5 ± 4.1	6.9 ± 0.9	0.038 ± 0.002	204.5 ± 11.6
	(*n* = 71)	(*n* = 71)	(*n* = 71)	(*n* = 61)	(*n* = 61)	(*n* = 61)	(*n* = 132)	(*n* = 132)
*Asterionellopsis glacialis*	0.357 ± 0.008	236.0 ± 5.5	1.12 ± 0.05	0.106 ± 0.002	70.1 ± 2.7	2.9 ± 0.1	0.051 ± 0.001	222.5 ± 5.3
	(*n* = 121)	(*n* = 121)	(*n* = 121)	(*n* = 59)	(*n* = 59)	(*n* = 59)	(*n* = 180)	(*n* = 180)
Other chain-forming diatoms 5–20 µm	0.211 ± 0.013p	122.9 ± 7.4	1.48 ± 0.14	0.054 ± 0.005	31.5 ± 3.0	5.5 ± 0.7	0.038 ± 0.002	145.0 ± 9.4
	(*n* = 53)	(*n* = 53)	(*n* = 53)	(*n* = 31)	(*n* = 31)	(*n* = 31)	(*n* = 104)	(*n* = 104)
**Dinoflagellates**								
*Tripos/Ceratium* spp.	0.015 ± 0.002	563.1 ± 59.4	9.98 ± 2.1	0.004 ± 0.001	169.6 ± 21.4	26.7 ± 2.3	0.015 ± 0.001	3013.7 ± 156.7
	(*n* = 46)	(*n* = 46)	(*n* = 46)	(*n* = 50)	(*n* = 50)	(*n* = 50)	(*n* = 96)	(*n* = 96)

Nitrogen (N)-specific ammonium assimilation (h^−1^), cell-specific ammonium assimilation (fmol cell^−1^ h^−1^), carbon-to-ammonium assimilation ratio, nitrogen-specific nitrate assimilation (h^−1^), cell-specific nitrate assimilation (fmol cell^−1^ h^−1^), carbon-to-nitrate assimilation ratio, carbon-specific carbon assimilation (average of ammonium and nitrate incubations), cell-specific carbon assimilation (average of ammonium and nitrate incubations, fmol cell^−1^ h^−1^), number of cells analyzed (*n*) from the ammonium (T5) and nitrate (T12) incubations in September. All values represent the average with the SE.

### Nitrogen assimilation and dynamics

**Figure 3:**
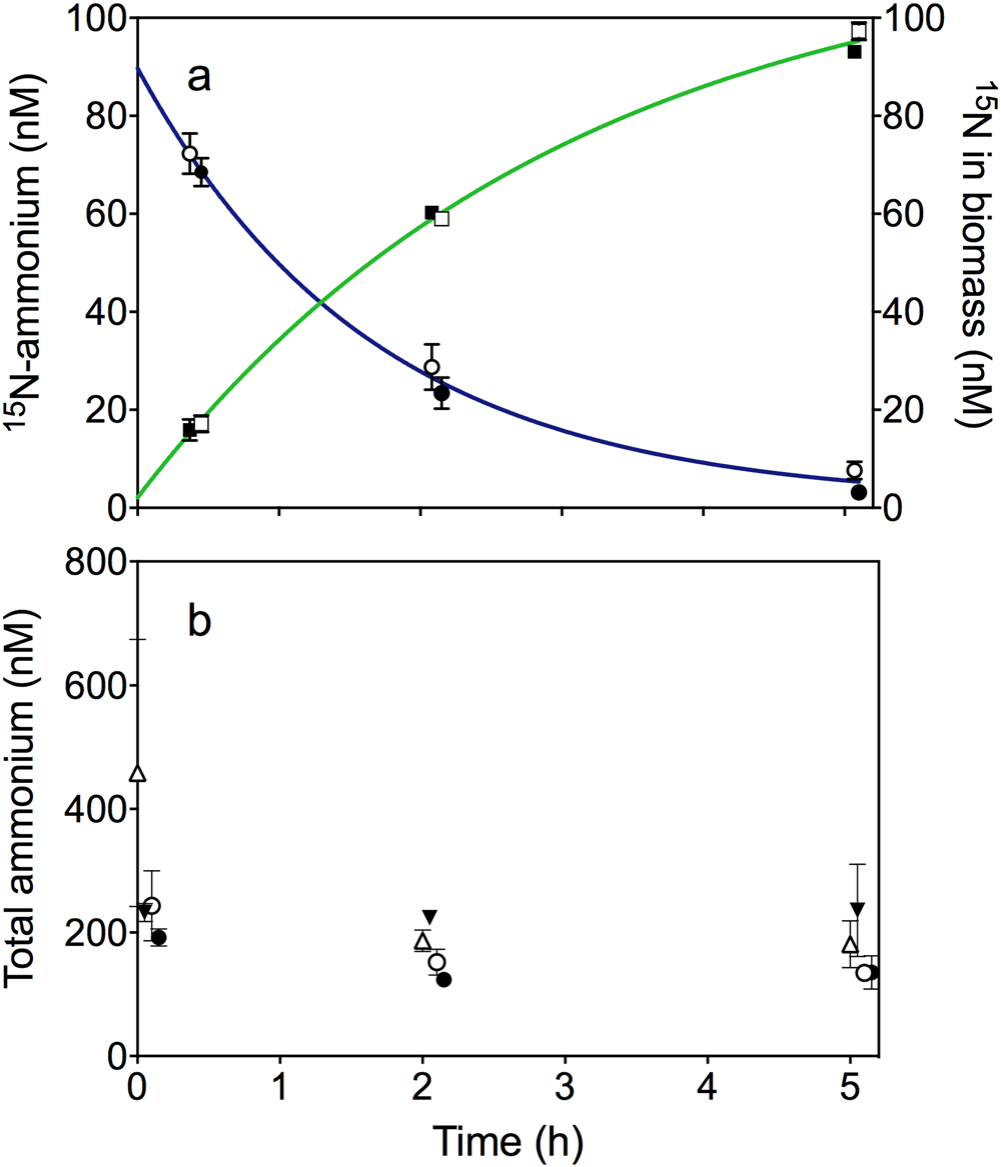
Ammonium dynamics. Panel a demonstrates the concentration of ^15^N ammonium dissolved in the water (circles, blue line), and assimilated into biomass (squares, green line), during September incubations. Closed symbols symbolize dark and open light incubations. Panel b demonstrates the total ammonium (^15^N and ^14^N) concentration (nM) in the flasks during the August (triangles) and September (circles) light (7:00 to 12:00, open symbols) and dark (19:00 to 24:00, closed symbols) incubations. Error bars indicate standard deviation, *n* = 3.

Diatoms assimilated nitrate in balance with C, but ammonium in surplus with C, according to Redfield (Table 1). In the dinoflagellates, the combined assimilation of DIN dominated by ammonium balanced their C growth according to Redfield ratio (Table 1). Up to 88% of total primary production in the phytoplankton community was based on ammonium as N source, with an overall POC:PON ratio close to Redfield (Supplementary Fig. S2 and Supplementary Table S4). We tracked the dynamics of ammonium assimilation in the mixed plankton community by adding ^15^N-labeled ammonium to the ambient water and followed its assimilation into particulate organic matter (POM) (Fig. 3a). Fifty to 70% of the ^15^N-labeled ammonium was assimilated and recovered as POM after 2 h, and 100% assimilated as POM after 5 h. However, the ambient concentration of total ammonium concentrations was at steady state at ca. 200 nM, indicating a balanced and tightly coupled production and consumption (Fig. 3b). Consequently, the availability of ammonium was poorly described by the low ambient concentration as it was continuously produced and consumed within the community with turnover times of a few hours. Production of ^15^N-nitrite or -nitrate from nitrification in ^15^N ammonium incubations was not detected.

### Assimilation of nitrate and ammonium into phytoplankton cells

**Figure 4:**
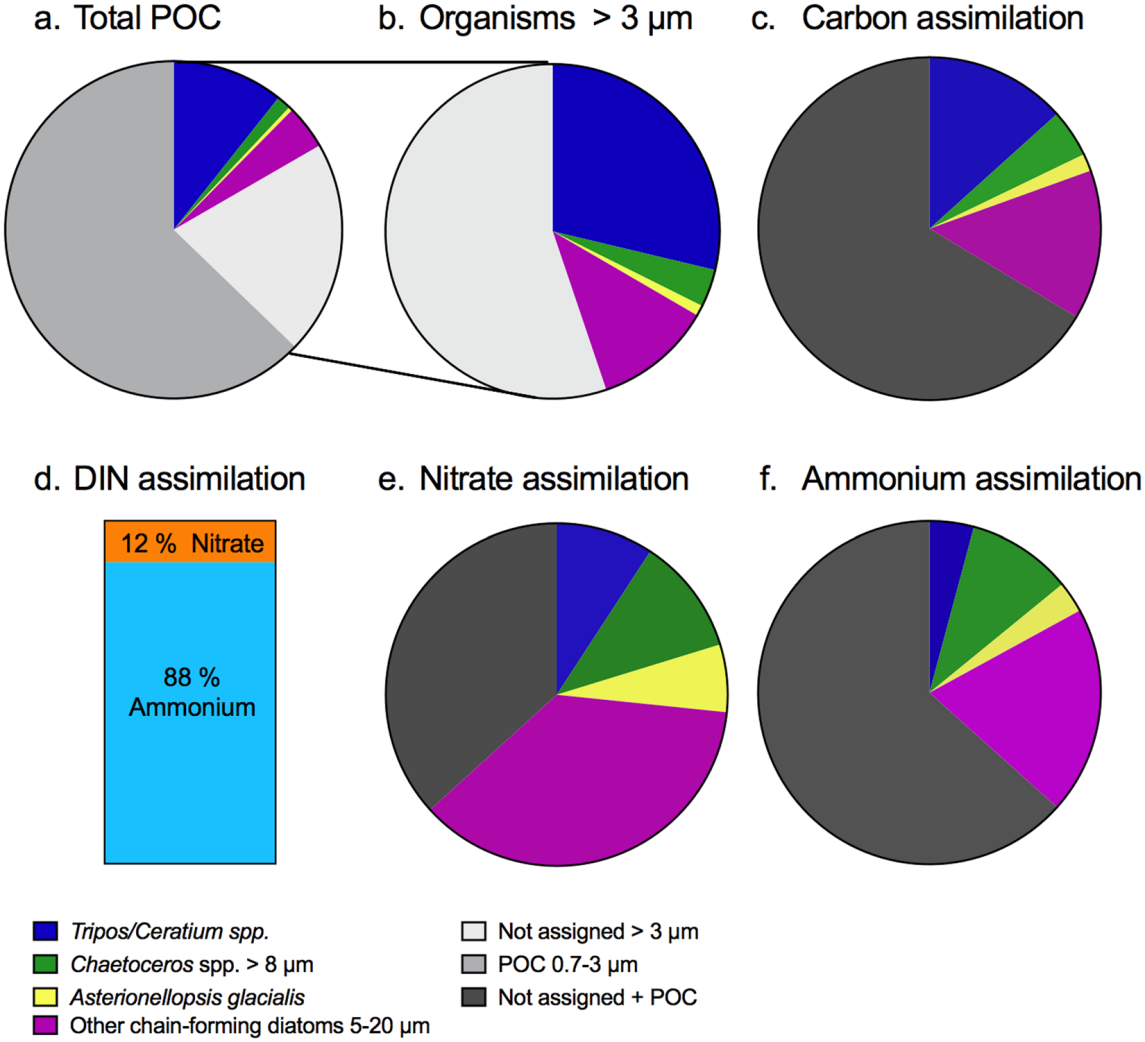
POC, C and N assimilation in the phytoplankton community. Relative particulate organic carbon (POC) >0.7 µm (**a**), identified organisms >3 µm (**b**), carbon assimilation (**c**), proportion between nitrate and ammonium assimilation (**d**), nitrate assimilation (**e**) and ammonium assimilation (**f**), during September incubations. Assimilation rates are noted for *Asterionellopsis glacialis*, other chain-forming diatoms 5–20 µm, *Chaetoceros* spp. >8 µm, *Tripos*/*Ceratium* spp., not assigned (65% dinoflagellates, 26% diatoms based on carbon biomass and not analyzed by SIMS), and not assigned + organisms >3 µm, which can either be not assigned organisms or POC between 0.7–3 µm analyzed on the GF/F filters.

Regeneration of ammonium within a turnover time of a few hours in the water supported 73 to 88% of total primary production (Figs. 4 and S2). The remaining 12% to 27% of total production was based on nitrate. The POC:PON ratio was close to the Redfield ratio (Table S4). Plankton cells >3 μm composed 37% of total POC and contributed approximately one-third of C assimilation in the plankton community (Fig. 4 and Supplementary Table S2). Even though the chain-forming diatoms only represented 6% of total POC, they contributed 20% of C assimilation, 54% of nitrate assimilation, and 32% of ammonium assimilation in the plankton community. C and nitrate assimilation by chain-forming diatoms to the community was higher than that by *Tripos*/*Ceratium* spp. due to higher abundance (cells L^−1^) of diatoms compared to *Tripos*/*Ceratium* spp. (Fig. 4). The large dinoflagellates represented 11% of the total POC, and contributed 14% of C assimilation, 4% of ammonium- and 9% of nitrate assimilation within the plankton community.

### Diffusion limitation of nutrient assimilation and cellular growth

Using mass transfer theory, we calculated the diffusion-limited supply of nitrate, ammonium, and phosphate to diatoms (Eq. 7) and to dinoflagellates (Eq. 8) from the ambient water. Here, *Chaetoceros* spp. exemplifies a chain-forming diatom genus and *Tripos/Ceratium* a dinoflagellate genus. The average cell-specific nitrate assimilation rate in *Chaetoceros* spp. was 42 ± 4 fmol N cell^−1^ h^−1^ (Table 1), thus, close to the average theoretical diffusion-limited supply of 37 fmol N cell^−1^ h^−1^. The measured cell-specific ammonium assimilation rate of 272 ± 12 fmol N cell^−1^ h^−1^ (Table 1), however, exceeded the average theoretical diffusion-limited supply of 62 fmol N cell^−1^ h^−1^ by 340% in *Chaetoceros* spp., which clearly indicates mechanisms other than diffusion from the ambient water to facilitate ammonium supply. The variation of the theoretical diffusion-limited supply of nitrate and ammonium to *Chaetoceros* spp. is related to variation in cell size and chain-length and was equal to ca. 43% (calculated based on minimum and maximum values divided by the theoretical diffusion-limited supply to cells and chains of average size). The diffusion-limited supply of nitrate and ammonium to large *Tripos/Ceratium* was 259 and 433 fmol N cell^−1^ h^−1^, respectively, while the measured cell-specific assimilation rates of nitrate and ammonium were 170 ± 21 and 563 ± 59 fmol N cell^−1^ h^−1^, respectively (Table 1). The error bound of the theoretical diffusion-limited supply for *Tripos/Ceratium* spp. related to variation in cell size and geometry was also equal to ca. 40%. While the average nitrate assimilation in *Tripos/Ceratium* was 34% lower than its average potential set by diffusion limitation, the average ammonium assimilation rate was 30% higher than the average diffusion-limited supply at the low, steady-state ammonium concentration in the ambient water. Thus, nitrate and ammonium assimilation rates in *Tripos/Ceratium* spp. were within the theoretical range for diffusion limited DIN supply. The average diffusion-limited supply of orthophosphate to cells was 6.5 and 45 fmol P cell^−1^ h^−1^ in *Chaetoceros* and *Tripos/Ceratium*, respectively. The ratios of measured total DIN uptake to diffusion-limited orthophosphate supply were 48 in *Chaetoceros* and 13 in *Tripos/Ceratium*. Hence, the N:P ratio was close to balanced growth relative to Redfield (N:P ratio of 16) in *Tripos/Ceratium* during diffusion-limited DIN assimilation and orthophosphate supply. In *Chaetoceros* cells, ammonium uptake was in surplus relative to diffusion-limited orthophosphate supply according to Redfield ratio, whereas the ratio of nitrate assimilation rate to diffusion-limited orthophosphate supply was 6.4 and thus close to Redfield ratio.

## Discussion

The physical, chemical, and biological constraints of cell-specific nutrient fluxes in large phytoplankton within mixed field communities are poorly known due to technical limitations. Diffusion limitation in large phytoplankton has previously been assessed by pure theoretical modelling and/or experimentally in monocultures^10–12^. Only recently, we introduced SIMS combined with stable isotopic tracers to assess diffusion limitation of DIN uptake and effects of turbulence in a diatom spring bloom population^7^ using a similar approach as herein. In the present study, we revealed nitrate and ammonium fluxes to large phytoplankton in a mixed phytoplankton field community during late summer with persistently low nutrient concentrations. We additionally compared the measured assimilation rates at a single-cell level with those predicted by mass transfer theory. The late summer light and nutrient conditions generated C-doubling times ranging from 1 to 3 days. Despite diffusion limitation of DIN assimilation in large phytoplankton cells, these genera contributed more than one third of total C assimilation in the community.

Dinoflagellates often use autotrophy and predation on other phytoplankton simultaneously to obtain C and nutrients, i.e., mixotrophy^18^. The C and N doubling times based on diffusion-limited supplies of DIN to large dinoflagellates observed herein suggest that these organisms must use mixotrophy to sustain their N demand under DIN limitation at higher growth rates than observed here. This is supported by a recent nanoSIMS laboratory study which demonstrated that the mixotrophic dinoflagellate, *Prymnesium parvum*, primarily relies on N from predation while C demand is covered by inorganic sources^19^. The nanoflagellate *Rhodomonas salina*, with a size of 6 to 7 μm, is a potential prey for dinoflagellates^11,20^ and contains approximately 550 fmol N cell^−1 ^^21^. Thus, capture and assimilation of a single flagellate per hour would provide as much N to the large *Tripos/Ceratium* as the measured N assimilation rate per hour in our study. Mixotrophy is likely the dominant lifestyle for these large dinoflagellates because of diffusion-limited uptake of inorganic nutrients. Our measurements within field communities support other recent laboratory studies demonstrating that swimming and feeding currents by mixotrophic dinoflagellates are needed to simultaneously increase nutrient uptake and interception of prey particles^11^. The present study, however, suggests that interception of prey is more important as N source as compared to DIN assimilation during swimming in field communities as the DIN fluxes from the ambient water only could support ca. two cell divisions per week.

Cell-specific nitrate assimilation rates in both diatoms and dinoflagellates as well as the ammonium assimilation rate in dinoflagellates were close to the values predicted by mass transfer theory. Recently, we found a similar match between predicted and measured cell-specific nitrate assimilation rate in *Chaetoceros* during a spring bloom when ambient nitrate concentrations were depleted below 0.3 μM concurrent with much lower ammonium assimilation rates by these diatoms^7^. In the present study of a summer population growing with persistently low DIN concentrations, however, the average cell-specific ammonium assimilation rate in *Chaetoceros* exceeded that predicted by diffusion limitation by more than 300%. Compared to nitrate, ammonium uptake is less energetically costly for the organisms, and its turnover rate is usually very high in the pelagic environment as also measured herein^7,14^. The classical mechanisms considered by which diffusion limitation can be alleviated in phytoplankton are advection by sinking (diatoms) and swimming (dinoflagellates). The effects of sinking and swimming on total uptake of nutrients in chain-forming diatoms and dinoflagellates are theoretically well studied and may, at most, double the total nutrient uptake relative to diffusion in large cells. This effect should be similar for nitrate and ammonium since their diffusion coefficients differ by less than 5%^12,22^. Turbulent strain is another important mechanism by which C and nutrient assimilation in chain-forming diatoms can be enhanced over diffusion by ca. 60% in the wave-breaking zone, but was not significant during our bottle incubations. The *in situ* dissipation rate of turbulent kinetic energy measured in the present study (Supplementary Material Fig. S1) corresponds to a turbulent strain rate of 1 s^−1^, which may increase the nutrient uptake in large *Chaetoceros* cell chains by only ca. 10% compared to that under stagnant conditions^7,11,12^. Thus, the ammonium uptake by far exceeded the classical mechanism considered to alleviate diffusion limitation of large phytoplankton. Patchiness of ammonium, which is the dominant excretion product among zooplankton and protists, may also alter the availability between phytoplankton cells^23^. However, this does not explain the large difference in ammonium assimilation between the chain-forming diatoms and dinoflagellates.

**Figure 5:**
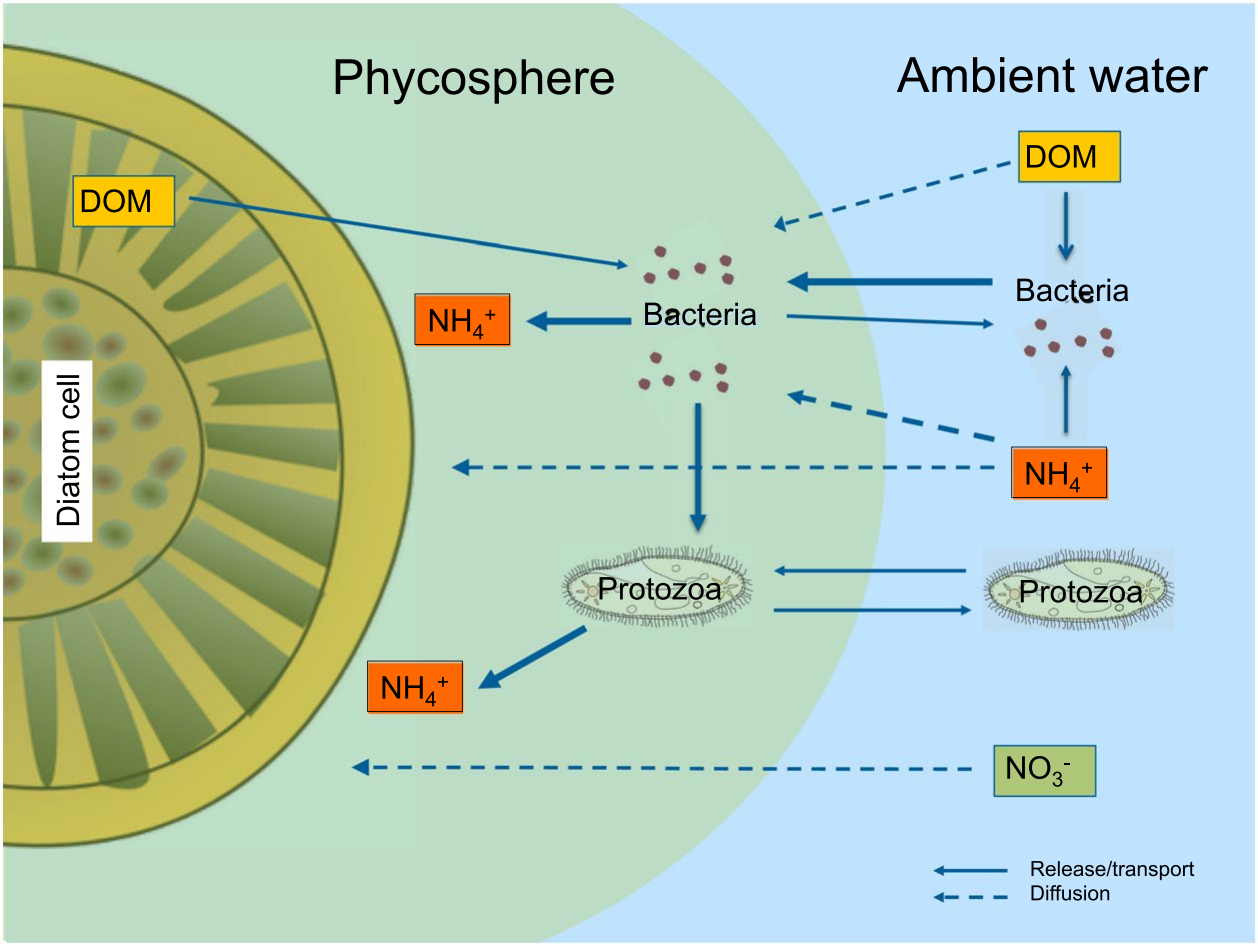
Microbial interactions and nutrient fluxes in the phycosphere. Bacteria colonize the phycosphere where they benefit from carbon-rich DOM released by diatoms and simultaneously remineralize N-rich DOM diffusing into the phycosphere from ambient water. The higher abundance of bacteria in the phycosphere, however, also attracts protozoa, which graze on the bacteria and release ammonium that is subsequently assimilated by the diatoms. Cartoons of organisms provided with the courtesy of the Integration and application network, University of Maryland Center for Environmental science (ian.umces.edu/symbols/).

Ammonium is the key inorganic nutrient produced during microbial degradation of dissolved organic matter (DOM) and during grazing of bacteria by protozoa (in this study e.g. ciliates and flagellates, Supplementary Information Table S1). *Chaetoceros* is known to release a large fraction of DOM during growth^24^, and several groups of bacteria can use chemotaxis, and thereby locate the enriched area around phytoplankton cells^13^. Bacteria are known to stimulate growth of diatom cells/chains suspended in cultures presumably due to nutrient recycling^25^. Field studies have shown that bacteria and protozoa are up to 10,000-fold more abundant on small diatom aggregates than in the ambient water^26^, and ammonium concentrations within diatom aggregates can be up to 100-fold higher than ambient concentrations^7,27^. Relatively stable chemical microenvironments measured by O_2_ and pH microsensors can be established within minutes to hours within and around millimeter small diatom aggregates as well as in other phytoplankton colonies^27–30^. Intense colonization of millimeter small model aggregates by motile bacteria also occurs within seconds to minutes and is strongly enhanced by chemical cues leaking from the aggregates, e.g., DMSP^31,32^. The fast ammonium transfer to *Chaetoceros* which exceeds the maximum theoretical fluxes from the ambient water by more than 300% documented in the present study may likely be facilitated by ammonium production during microbial interactions at close proximity between diatoms and microbes, i.e., within the phycosphere (Fig. 5). Unfortunately, it is not possible to measure ammonium concentration gradients directly in the phycospheres of single cells in sea water because of salt interference in ammonium microsensors^33^. The measurement of bacterial abundance and microbial interactions directly in the phycosphere is also challenging. However, we propose that *Chaetoceros* maintain its own source of nutrients through bacterial recycling within their phycosphere under fluctuating conditions. Bacterial remineralization of DOM from the diatom cell simultaneously with nitrogen-rich DOM from the ambient water can facilitate net N transfer from ambient water to the phycosphere of diatoms. Grazing of bacteria by protozoa within the phycosphere would also contribute to ammonium production and transfer to diatoms. In a scenario with 100-fold higher bacterial abundance in the phycosphere compared to that in the ambient water, a colonization and grazing loss rate of 0.027 h^−1 ^^26^ and a 50% release of ammonium during protozoan grazing by protozoa would produce 200 fmol N h^−1^ within the phycosphere of each diatom cell. This production matches the measured surplus of cell-specific ammonium assimilation rates relative to the diffusion-limited supply in *Chaetoceros* (for details please see Supplementary information). A lower bacterial abundance but higher colonization and grazing rates could produce a similar ammonium production within the phycosphere. The cell-specific ammonium assimilation rates of the other two groups of chain-forming diatoms were similar high as those of *Chaetoceros*, and diffusion-limitation may thus be alleviated in a similar way as *Chaetoceros*.

Isotopic images of ^15^N:^14^N showed enrichment of ^15^N in the vicinity of *Chaetoceros*, but cannot be used quantitatively to assess bacterial and protozoan abundance in the phycospheres (Fig. 1). However, the isotopic ratio images of ^13^C:^12^C and ^15^N:^14^N in *Chaetoceros* of this and our previous study^7^ clearly showed that freshly assimilated ^13^C, but not ^15^N nitrate or ^15^N ammonium, was associated with spines after incubations with ^13^C bicarbonate in light during 5 to 12 h. Some *Chaetoceros* species, e.g., *C. danicus, C. castracanei, and C. concavicornis*, contain chloroplasts in their spines^34,35^. These species, however, comprised a very small fraction of the biovolume of *Chaetoceros* species which was clearly dominated by *C. affinis* (Fig. 1; Supplementary Table S2) that does not contain chloroplasts in its spine. Thus, this organic matter enriched in ^13^C associated to the spines of *C. affinis* (Fig. 1) may be exudates. Recent studies using digital holographic microscopy (D^3^HM) have shown that TEP is associated to the spines of *Chaetoceros* (Zetsche and Ploug, pers. Com.). Freshly assimilated ^15^N nitrate and ^15^N ammonium was detected directly within the cells as CN compounds by SIMS. While assimilation of C:nitrate:orthophosphate was close to Redfield ratio, ammonium was assimilated in surplus. Hence, luxury uptake of ammonium was presumably efficiently transformed and stored as DON, e.g., urea, in the vacuole of *Chaetoceros* for later use or excretion in an environment with fluctuating nitrate concentrations^36^. Urea with its low C:N ratio is a likely storage product, which was recently demonstrated to be substantial in diatoms and has been suggested as a key to their success under variable conditions^37–39^.

While ammonium was recycled within the surface water, nitrate was presumably imported into the euphotic zone across the thermocline as no production of ^15^N nitrite or nitrate from nitrification in ^15^N ammonium incubations was detected. New primary production based on nitrate, e.g., by diatoms, is predicted to decrease during climate change due to increased warming and stratification of the surface ocean, which limit nitrate import across the thermocline to the euphotic zone^40,41^. The present study demonstrates that production by chain-forming diatoms can be higher than anticipated where diatom abundance and nutrient concentrations are low due to their alternative strategies to maintain high nutrient transfer within their phycosphere. The C and N growth rates in *Chaetoceros* were as high as those measured during the exponential growth phase in cultures of other chain-forming diatoms using similar methods as herein^42^. The diffusion-limited nitrate assimilation rate matched the C assimilation rate according to Redfield ratio even when ammonium was available and concurrently assimilated by the diatoms. Thus, the chain-forming diatoms were the key organisms mediating 20% of total primary production and assimilating 54% of the nitrate and 32% of the ammonium in the mixed phytoplankton community despite their low contribution of 6% to the total POC in the plankton community. High cell-specific growth rates but a low standing stock of diatoms must be counter balanced by high loss rates and are likely explained by zooplankton grazing and sedimentation^5,7,43^. Consequently, new production by chain-forming diatoms can be high despite low cellular abundance and low steady-state DIN concentrations within plankton communities.

## Methods

### Water sampling and stable isotope incubations

Seawater was collected using a water sampler (ca. 1.7 L, Ruttner, Heraco AB) near a monitoring buoy (N 58°25′63, E 11°45′14), situated at the entrance of the Gullmar Fjord, on the Swedish west coast. The water was collected at 5 m (August 22^nd^, 2017) and 3 m (September 19^th^, 2017) depth, in the morning (5:30) and in late afternoon (17:30). Water temperature was 18 °C and 16 °C in August and September, respectively, with an average salinity of 24, which was measured by a conductivity meter (Table S5).

The collected water was gently poured into 1.1 L bottles within one hour of collection. Each bottle was amended with stable isotopic tracers to final excess labeling of 3.9–4.3% (^13^C bicarbonate, Sigma), 21–36% (^15^N ammonium, Sigma) and 18–41% (^15^N nitrate, Sigma). Three bottles were directly sacrificed for the initial (0 h) time point and three bottles of each treatment were hung horizontally *in situ* at 5 m depth in August and 3 m depth in September, for 2 and 5 h for ^15^N ammonium incubations and 12 and 24 h for ^15^N nitrate incubations. Average light at respective incubation depths at noon was around 120 µmol photons m^−2^ s^−1^ in August and around 50 µmol photons m^−2^ s^−1^ in September (converted from lux^44^, measured *in situ* by HOBO loggers). Time of sunrise and sunset is given in Table S5. Water collected in late afternoon was used for evening incubations with ^15^N ammonium, for 0, 2 and 5 h (Table S5). At each time point, 300–500 mL of each incubation replicate was stopped by filtration under low pressure (<200 mbar) onto precombusted (4 h, 450 °C) GF/F filters (0.7 μm). Filters were dried overnight (60 °C), de-calcified in HCl fumes in a desiccator overnight, and packed into tin cups for later analysis of ^15^N and ^13^C incorporation into biomass.

### Turbulence and density measurements in the water column

Profiles of temperature, salinity, pressure, and microstructure shear were obtained with a semi-free-falling microstructure profiler (MSS90L) dropped from the side of a small drifting vessel near the water intake at Sven Lovén Centre for Marine Sciences, Kristineberg. A total of 17 profiles were obtained on 22 August 2017 in three ensembles during the time intervals 13:47–14:06 (ensemble 1), 14:45–14:57 (ensemble 2), and 15:22–15:30 (ensemble 3). Microstructure shear was measured with two airfoil shear probes (PNS06) sampled at 1024 Hz, and dissipation rates of turbulent kinetic energy was estimated from the microstructure shear as described in further detail in Arneborg and Liljebladh^45^. The wind speed where 2–6 m s^−1^ in August during daytime (4–5 m s^−1^ when the *in situ* turbulence was measured) and 3–6 m s^−1^ in September during daytime. The wind direction were 160–220° in August and 290–320° in September. The wind speed is directly related to turbulence in the water^46^, and thus, equal to a shear rate of ca. 1 s^−1^ at 5 m during both experiments.

### Dissolved inorganic nutrients

Immediately following each water collection, five sub-samples were pre-filtered (0.2 µm) and frozen until analysis for dissolved inorganic nutrients (ammonium, nitrite and nitrate, silicate, and phosphate). Nutrients were measured at Sven Lovén Centre for Marine Sciences, Kristineberg^47^. Total ammonium concentrations were monitored throughout the time series incubations immediately after water filtration^48^. Samples with filtrate for dissolved inorganic carbon (DIC) were fixed with ZnCl_2_ (100 µL 50% w/v) in 12 mL exetainers (Labco, UK) and were stored at room temperature until analysis. Samples to determine the ^15^N composition in ammonium, nitrate and nitrite were filtered (0.2 µm) and frozen until analysis.

### Microscopic analyses of community composition

At each time point, 50 ml of each replicate bottle was collected to identify and count organisms >3 μm by preservation with alkaline Lugol’s solution and stored dark at ca. 4 °C until analysis^49,50^. The genera *Tripos* and *Ceratium* were grouped together in the single cell analysis (SIMS), due to their close relationship and newly revised lineage^51,52^. Lengths and widths of the organisms were measured in order to calculate their biovolumes (mm^3^ L^−1^). Biovolumes were used to calculate concentrations of POC (µM) according to Menden-Deuer and Lessard^21^.

### Isotopic analyses

POC and PON as well as incorporation of ^15^N nitrate, ^15^N ammonium and ^13^C bicarbonate were analyzed by Elemental Analysis Isotope ratio mass spectrometry (EA-IRMS) at the Department of Geology, University of Gothenburg. The isotopic composition of dissolved nitrate, nitrite, ammonium, and DIC were measured via gas chromatography isotope ratio mass spectrometry (GC-IRMS) at the University of Southern Denmark (SDU, Odense). Concentrations of ^15^N ammonium were measured by conversion of ammonium to N_2_ with alkaline hypobromite iodine^53^. Potential production of ^15^N nitrite from nitrification was determined by conversion of nitrite to N_2_ with sulfamic acid^54^. Concentrations of ^15^N nitrate were determined by removal of nitrite from the sample with sulfamic acid, conversion of nitrate to nitrite with cadmium, and final conversion of nitrite to N_2_^54,55^. Analysis of ^15^N in N_2_ (^29^N_2_, ^30^N_2_) was determined by GC-IRMS (Thermo Delta V Plus)^56^. For analysis of ^12^C and ^13^C DIC, 600 µL of sample were acidified with 50 µL 85% phosphoric acid in a helium-flushed exetainer to convert DIC to CO_2_. The GC-IRMS was modified to bypass the copper reduction column, and CO_2_ traps and gas samples were introduced as before. Concentrations of ^12^CO_2_ and ^13^CO_2_ were used to calculate labeling percentages in DIC.

### Secondary ion mass spectrometry

Samples of 45 mL were collected by pouring slowly into falcon tubes, preserving with 1–2% paraformaldehyde (PFA), and stored dark at 4 °C for 24 h. They were thereafter gently filtered (<200 mbar) onto TTTP filters (2 μm), washed with phosphate buffered saline (PBS, 10X, pH 7.4), and stored at room temperature until analyzed. ^13^C and ^15^N incorporation into single cells was analyzed using SIMS on an IMS 1280 (Cameca, Genneviliers, France) at the Natural History Museum in Stockholm, Sweden. The TTTP filters were cut into ca. 4 × 4 mm pieces and glued onto glass slides, and thereafter coated with a thin (ca. 10 nm) layer of gold. Areas of interest (100 × 100 for diatoms and 140 × 140 µm for dinoflagellates) on the filters were pre-sputtered with a primary cesium-ion (^133^Cs^+^) beam (3 or 10 nA, with the stronger used for the diatoms to remove the silica frustule) for 240–300 s and then using a 100 pA Cs^+^ beam with a spatial resolution of ca. 1 µm for 60 cycles of imaging. Secondary ion images (265 × 265 pixels) were recorded for ^12^C^15^N^−^, ^13^C^14^N^−^ and ^12^C^14^N^−^ using a peak-switching routine at a mass resolution of 12 000 (M/ΔM) and an ion-counting electron multiplier. Imaging and data processing were done using the Cameca WinImage2 software. Individual cells were defined as regions of interest (ROIs) based on cell morphology on ^12^C^14^N^-^ images from which the cell-specific isotope ratios ^13^C/^12^C and ^15^N/^14^N were calculated. Each individual image was carefully examined by eye and the ROI was defined along the cell border and drawn by hand. Increasing numbers of the cellular C and N assimilation rates measured by SIMS were calculated until the excess^13^C/^12^C and excess ^15^N/^14^N per cell were stable and the standard error of calculated assimilation rates was <10% of the average value (please see below). Hereby, we achieved representative average values for single cells^7^ of *Chaetoceros*, other chain-forming diatoms, and *Tripos/Ceratium* to calculate their respective contributions to community C and N assimilation rate.

### C and N assimilation rates

The total C assimilation rates (nM h^−1^) in the plankton community were calculated from the ^13^C-atom% excess of DIC in the ambient water and the change in excess isotopic composition of organic matter during the incubation time, t^57^:

1 $$C\,assimilation\,rate=\frac{{\Delta }13C-atom \% \,exces{s}_{(POC)}\times POC}{13C-atom \% \,exces{s}_{(DIC)}\times {\Delta }t}$$

Cell-specific C assimilation rates were calculated analogously from the average POC content of single cells^21^ and the change in ^13^C-atom% excess isotopic composition of single cells during the incubation time, t, as done previously^7^.

No nitrification was detected during the incubations. Thus, nitrate assimilation rates were calculated from the ^15^N-atom% excess of nitrate in the ambient water and the change in excess isotopic composition of organic matter during the incubation time, t^57^:

2 $$Nitrate\,assimilation\,rate=\frac{{\Delta }15N-atom \% \,exces{s}_{(PON)}\times PON}{15N-atom \% \,exces{s}_{(nitrate)}\times {\Delta }t}$$

Cell-specific N assimilation rates at a single-cell level were calculated analogously from average PON content of single cells^21^ and the change in ^15^N-atom% excess isotopic composition of single cells during the incubation time, t, as done previously^7^.

The ^15^N ammonium-atom% excess depends on regeneration of ^14^N ammonium during the incubation. The ^15^N:^14^N ratio decreases exponentially in the ambient water during first-order kinetics concurrent with dilution of the ^15^N from regenerated ^14^N. Assimilation of ammonium was calculated based on the average excess atom% during the incubation time, R^58^:

3 $$R=\frac{{R}_{0}}{kt}[1-\exp (kt)]$$

where *R*_0_ is the initial ^15^N ammonium-atom% excess, t is the incubation time and k is the specific decrease in ^15^N ammonium-atom% excess per unit time^58^:

4 $$k=\frac{\mathrm{ln}\,({R}_{t}/{R}_{0})}{t}$$

Ammonium assimilation rates (nM h^−1^) were calculated based on the average ^15^N ammonium-atom% excess, R, during the incubation time (t)^58^:

5 $$Ammonium\,assimilation\,rate=\frac{\Delta 15N-atom \% \,exces{s}_{(PON)}\times PON}{R\times \Delta t}$$

Cell-specific ammonium assimilation rates were calculated analogously from the average PON content of single cells^21^ and the change in ^15^N-atom% excess isotopic composition of single cells and the average ^15^N ammonium-atom% excess, R, during the incubation time, t.

### C and N growth rates at a single-cell level

The C growth rate and the N growth rates (h^−1^) based on nitrate and ammonium, respectively, were calculated assuming an even distribution of the isotope in the biomass during cell division^42^:

6 $$C\,or\,N\,based\,growth\,rate\,({h}^{-1})=\frac{atom \% \,exces{s}_{(POM)}}{atom \% \,exces{s}_{(dissolved)}}\times 2\times 1/t$$

where atom% excess_(POM)_ represents the excess isotope ratio of organic matter, atom% excess_(dissolved)_ represents the excess isotope ratio of DIC, nitrate or ammonium during the incubation, and *t* is the incubation time. The factor of 2 describes the dilution of the heavy isotope during assimilation into organic matter with an original isotope composition equal to the natural background during cell division. C and N doubling times were calculated as ln2/k, assuming exponential growth.

### Mass transfer

*Chaetoceros* spp. is an example of a chain-forming diatom genus because of its regular geometry. The potential diffusive inorganic N-supply to *Chaetoceros* cells within average cell chains (10 μm in diameter, 23 μm cell length and 7.9 cells chain^−1^) were calculated from the analytical solutions of diffusion to a cylinder under still conditions^59^:

7 $${Q}_{t}=[8+6.95{(\frac{L}{D})}^{0,76}]{r}_{0}D({C}_{\infty }-{C}_{0})$$

where *Q*_*t*_ is the quantity of substance diffusing to a cell within the chain with the length *(L)* and diameter *(D)* per unit time, *t*, *D* is the diffusion coefficient of the substance, *C*_0_ its concentration at the cell surface, *r*_0_, and *C*_*∞*_ the concentration in the ambient water far from a cell. The equation is considered accurate for L/D < 8. We assumed *C*_0_ to be zero, and used molecular diffusion coefficients of 1.53 × 10^−5^ cm^2^ s^−1^ for nitrate, of 1.60 × 10^−5^ cm^2^ s^−1^ for ammonium and of 5.73 × 10^−6^ cm^2^ s^−1^ for orthophosphate at 18 °C and a salinity of 24 as previously reported^22^. During our incubations with stable isotopic tracers, the *C*_*∞*_ was 0.15, 0.24, and 0.07 μM for nitrate, ammonium and orthophosphate, respectively.

Dinoflagellates were represented by *Tripos/Ceratium*. The potential diffusive inorganic N and phosphorus supplies to *Tripos/Ceratium* cells were calculated assuming spherical geometry of the major biomass of these cells (50 μm diameter of the bulk biomass) according to^60^:

8 $${Q}_{t}=4\pi D{r}_{0}({C}_{\infty }-{C}_{0})$$

## Supplementary information

Supplementary information

## Data Availability

All data generated or analyzed during this study are included in this article and its Supplementary Information file. Raw data are available form the corresponding author on upon request.
