# Supplementary material for: Nitrate and ammonium fluxes to diatoms and dinoflagellates at a single cell level in mixed field communities in the sea
Source: Sci Rep. 2019 Feb 5;9:1424. doi: 10.1038/s41598-018-38059-4 (PMC6363804; doi:10.1038/s41598-018-38059-4)
Supplement: Supplementary file 1 — Supplementary information [file 41598_2018_38059_MOESM1_ESM.pdf]

Supplementary information – Additional calculations, graphs and tables

"Nitrate and ammonium fluxes to diatoms and dinoflagellates at a single cell level in mixed field communities in the sea"

Malin Olofsson[1,*], Elizabeth K Robertson[1], Lars Edler[2], Lars Arneborg[1,3], Martin J Whitehouse[4], Helle Ploug[1]

[1]Department of Marine Sciences, University of Gothenburg, SE 405 30, Gothenburg, Sweden

[2]WEAQ AB, Doktorsgatan 9D, Ängelholm, Sweden

[3]Research and Development Department, Swedish Meteorological and Hydrological Institute, Norrköping, Sweden

[4]Swedish Museum of Natural History, Stockholm, Sweden

[*]Corresponding author:

E-mail: malin.olofsson@marine.gu.se

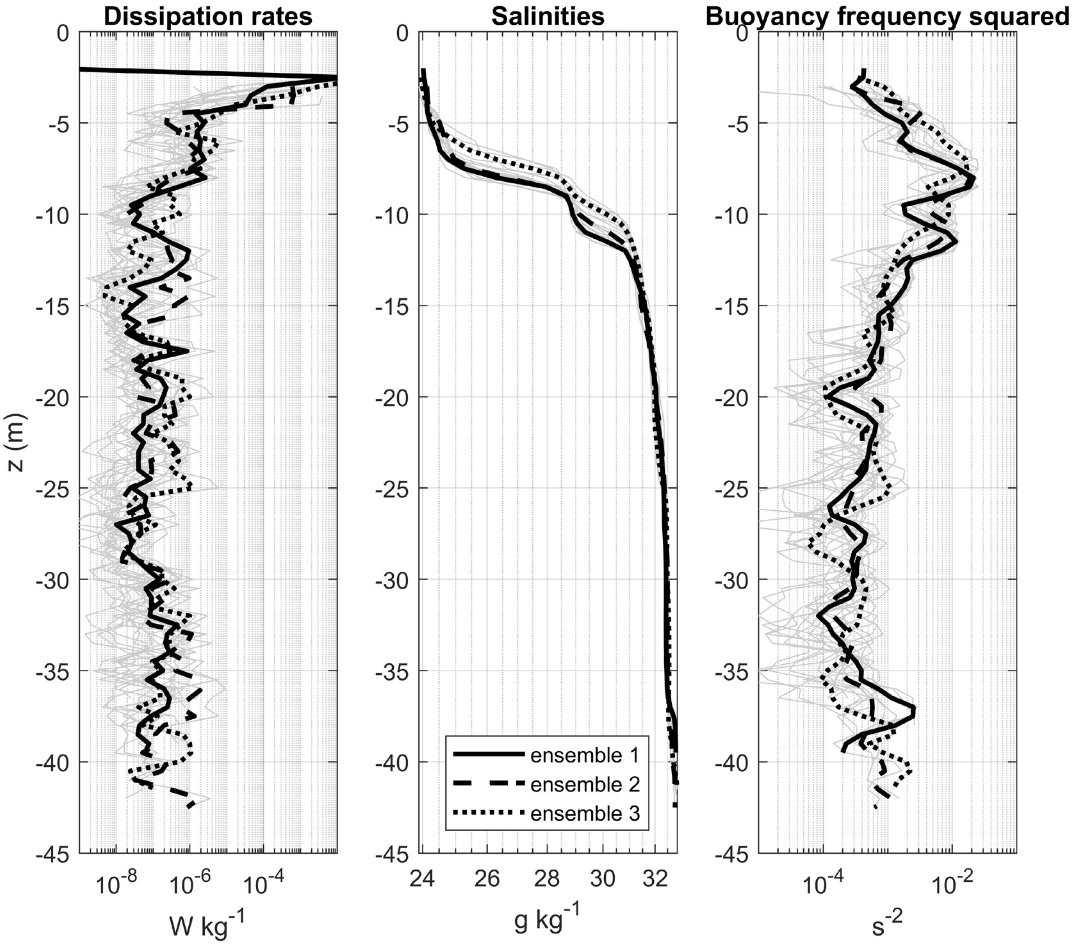

Figure S1. Turbulence measurements in situ in the Gullmar Fjord. Three depth profiles (m) where the left panel shows dissipation rates (W/kg), the middle panel salinities (g/kg) and the right panel buoyancy frequency squared (s^{-2}). Thick lines show ensemble averages and thin grey lines show individual profiles. Note that dissipation rate estimates shallower than 5 m depth are likely too large due to influence from vessel movements.

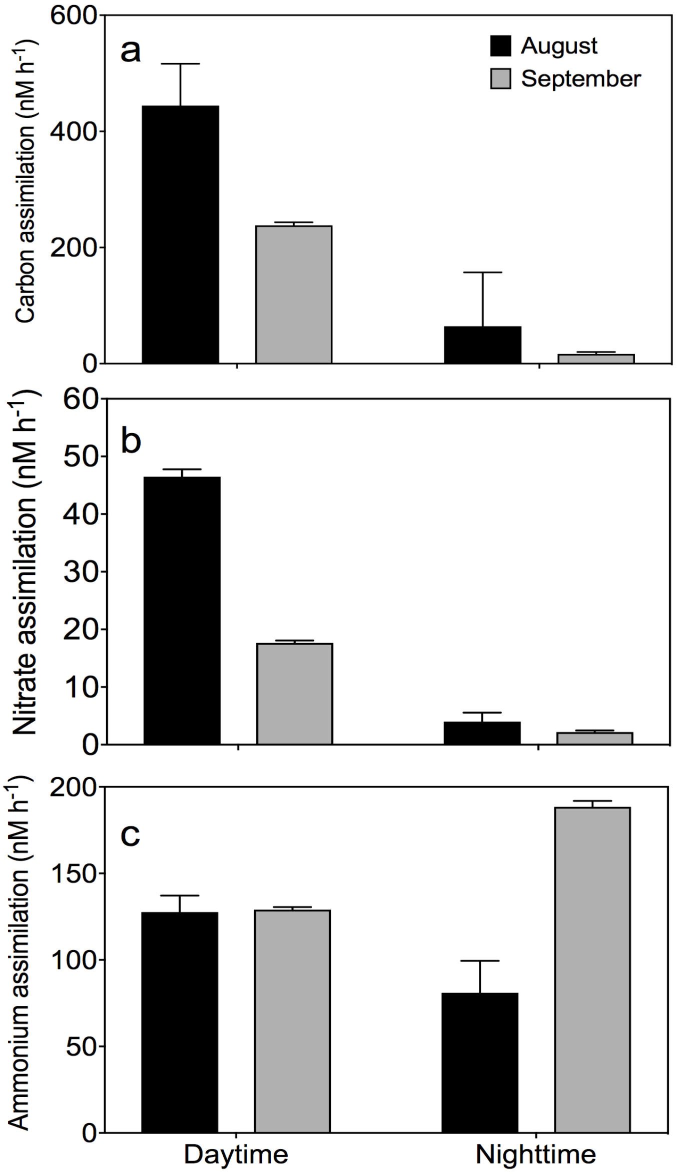

Figure S2. Community carbon (a), nitrate (b) and ammonium (c) assimilation rates (nM h^{-1}) in August and September during day (7:00 to 19:00) and night (19:00 to 7:00) for carbon and nitrate, and light (7:00 to 12:00) and dark (19:00 to 24:00) for ammonium. Error bars indicate standard deviation, n = 3.

Table S1 Identified taxa (> 3 µm) during light and day incubations in September. The table includes biovolume ($mm^3 L^{-1}$) per taxa, average cell width (µm) and length (µm), average volume per cell ($µm^3$), and carbon (C) and nitrogen (N) content ($pmol\ cell^{-1}$). Bold text mark the assigned taxa for SIMS analysis, either as single species or group of species including *Asterionellopsis glacialis*, *Chaetoceros* spp. > 8 µm, *Tripos/Ceratium* spp. or other chain-forming diatoms 5-20 µm.

Taxa	Total biovolume ($mm^3 L^{-1}$)	Width (µm)	Length (µm)	Biov. $cell^{-1}$ ($µm^3$)	C (pmol $cell^{-1}$)	N (pmol $cell^{-1}$)
Asterionellopsis glacialis	0.023	8-12	70-100	1434	8.7	1.3
Attheya septentrionalis	<0.001	3-4	8-10	64	0.7	0.1
Cerataulina pelagica	0.217	5-20	30-100	4479	21.9	3.3
Chaetoceros spp.	0.008	5-15	6-15	260	2.2	0.3
Chaetoceros affinis	0.034	8-18	15-22	1847	10.7	1.6
Chaetoceros castracanei	<0.001	18-20	18-20	4084	20.4	3.1
Chaetoceros ceratosporus	0.004	6-10	9-13	309	2.5	0.4
cf. *Chaetoceros circinalis*	0.001	15-25	10-14	3203	16.7	2.5
Chaetoceros concavicornis	<0.001	12-16	10-14	317	2.6	0.4
Chaetoceros contortus	0.003	6-12	12	604	4.3	0.7
cf. *Chaetoceros costatus*	<0.001	15-20	15	2705	14.6	2.2
Chaetoceros curvisetus	0.001	15-30	15-20	2592	14.1	2.1
Chaetoceros danicus	<0.001	10	11	633	4.5	0.7
Chaetoceros debilis	0.010	11-20	15	2260	12.6	1.9
Chaetoceros decipiens	0.006	15-30	11-21	6850	31.0	4.7
Chaetoceros diadema	0.003	15-40	20-27	12910	51.8	7.8
Chaetoceros didymus	0.021	10-35	10-25	7468	33.2	5.0
Chaetoceros lorenzianus	<0.001	20-25	15-25	4239	21.0	3.2
Chaetoceros minimus	<0.001	1-2	5-7	11	0.2	0.0
Chaetoceros pseudocrinitus	0.001	10-15	10-15	1150	7.3	1.1
Chaetoceros similis	0.001	8-13	12-17	977	6.4	1.0
Chaetoceros socialis	0.004	5-9	5-11	162	1.5	0.2
Chaetoceros subtilis	0.001	3-4	8-17	84	0.9	0.1
Chaetoceros tenuissimus	<0.001	3-4	3-9	52	0.6	0.1
Chaetoceros throndsenii	<0.001	3	6-14	62	0.7	0.1
***Chaetoceros* spp. > 8 µm**	0.087	8-40	8-25	1850	10.7	1.6
Cocconeis sp.	0.001	17	29	2322	12.9	2.0
Coscinodiscus spp.	0.003	90-130	90-130	196250	470.6	71.3
Coscinodiscus radiatus	0.003	70-90	90-90	351680	755.3	114.4
Cylindrotheca closterium	0.001	<3-5	20-35	139	1.3	0.2
Dactyliosolen blavyanus	0.076	15-30	100-150	49676	154.4	23.4
Dactyliosolen fragilissimus	0.068	5-30	30-100	7248	32.4	4.9
Diatoma tenuis	<0.001	6	30-50	427	3.3	0.5
Ditylum brightwellii	0.050	15-30	80-200	52674	162.0	24.5
Eucampia zodiacus	0.006	10-80	20-50	12385	50.1	7.6
Guinardia delicatula	0.044	10-15	30-50	3555	18.2	2.8
Leptocylindrus danicus	0.056	5-11	35-60	1917	11.0	1.7
Leptocylindrus minimus	0.005	3	20-50	201	1.8	0.3
Licmophora sp.	0.005	15-45	30-65	81000	229.6	34.8
Navicula sp.	0.001	10	30-40	1399	8.5	1.3
Nitzschia longissima	0.003	3-6	85-155	329	2.6	0.4
Pennales (spp.)	<0.001	4-6	10-25	113	1.1	0.2
Proboscia alata	0.001	7-10	250-500	31521	106.8	16.2

Pseudo-nitzschia spp.	0.008	3-4	70-100	612	4.4	0.7
Pseudo-nitzschia delicatissima GRP	0.003	1-2.5	50-70	146	1.4	0.2
Pseudo-nitzschia seriata GRP	0.033	5-8	80-110	3083	16.2	2.5
Pseudosolenia calcar-avis	0.041	12-25	300-500	98887	269.9	40.9
Rhizosolenia hebetata	0.004	3-7	130-400	2239	12.5	1.9
Rhizosolenia imbricata	<0.001	10-15	230-260	30051	102.7	15.6
Rhizosolenia setigera	0.006	4-20	140-450	37089	121.9	18.5
Skeletonema marinoi	0.080	3-10	7-25	520	3.8	0.6
Striatella unipunctata	0.002	65	60	61230	183.0	27.7
**Other chain-forming diatoms 5-20 µm**	0.140	5-20	5-60	1234	7.7	1.2
Thalassionema sp.	<0.001	3-5	110-120	1227	7.7	1.2
Thalassionema nitzschioides	0.001	2-5	40-80	978	6.4	1.0
Thalassiosira sp.	0.003	32-40	40-50	17608	66.6	10.1
Thalassiosira angulata	<0.001	30-35	30-35	12935	51.9	7.9
Thalassiosira gravida	0.001	20-37	20-37	9302	39.7	6.0
Akashiwo sanguinea	0.007	40	60-80	34062	326.3	49.4
Alexandrium ostenfeldii	0.003	50	50-55	70650	593.0	89.8
Ceratium/Tripos furca	0.219	35-55	35-55	30105	294.9	44.7
Ceratium/Tripos fusus	0.006	15-35	15-35	16795	182.8	27.7
Ceratium/Tripos horridum	0.001	35-45	35-45	24726	251.0	38.0
Ceratium/Tripos lineatum	0.004	25-35	30-35	8239	102.0	15.5
Ceratium/Tripos macroceros	0.001	40-50	40-50	35403	336.7	51.0
Ceratium tripos	0.207	50-70	50-70	82364	672.4	101.9
***Ceratium/Tripos* spp.**	0.438	15-70	15-70	45592	414.2	76.1
Dinophyceae spp.	0.002	20-25	20-25	5961	78.3	11.9
Dinophysis acuminata	0.019	48-52	48-52	24124	246.0	37.3
Dinophysis norvegica	0.003	60-70	60-70	46493	420.9	63.8
Dinophysis tripos	0.004	75	75	37680	354.4	53.7
Gonyaulax digitale	0.002	36	56	25095	254.0	38.5
Gonyaulax spinifera	<0.001	40	40	25120	254.2	38.5
Gymnodiniales spp.	0.105	15-50	15-70	6116	79.9	12.1
Gymnodinium spp.	0.010	11-42	15-55	1940	31.2	4.7
Gyrodinium spp.	0.004	11-50	15-65	3430	49.8	7.5
Gyrodinium spirale	0.001	22-32	70-100	15260	169.0	25.6
Heterocapsa rotundata	<0.001	5-7	10-12	132	3.5	0.5
Heterocapsa triquetra	<0.001	15	22-24	1354	23.3	3.5
Karenia mikimotoi	<0.001	26-30	26-30	6404	83.0	12.6
Katodinium glaucum	0.003	14-25	31-36	12473	143.3	21.7
Lingulodinium polyedrum	0.048	37-41	44-50	33938	325.3	49.3
Oblea rotunda CPX	0.004	22-28	22-28	7359	93.0	14.1
Peridiniales (spp.)	0.010	10-20	10-27	4723	64.7	9.8
Peridiniella danica	<0.001	18-22	18-22	2721	41.2	6.2
Phalacroma rotundatum	<0.001	45-55	45-55	14719	164.1	24.9
Polykrikos schwartzii	0.061	40-70	80-130	653905	3668.9	555.9
Prorocentrum micans	0.045	30-40	50-60	21720	225.7	34.2
Prorocentrum triestinum	0.003	10-15	25-30	1119	19.9	3.0
Protoceratium reticulatum	0.004	35-45	35-45	30144	295.2	44.7
Protoperidinium sp.	<0.001	35	40	13823	155.9	23.6
Protoperidinium brevipes	<0.001	23-29	23-29	6899	88.2	13.4
Protoperidinium claudicans	0.001	55	83	87465	706.3	107.0
Protoperidinium conicoides	0.002	44-57	44-57	58528	508.3	77.0
Protoperidinium curtipes	0.001	65-75	65-95	134628	1005.5	152.4
Protoperidinium crassipes	0.001	70	95	85264	691.7	104.8
Protoperidinium depressum	0.002	65-105	95-115	314000	2012.0	304.8

Protoperidinium divergens	0.060	60-65	80-85	83737	681.6	103.3
Protoperidinium oblongum	<0.001	50	85-95	60183	520.0	78.8
Protoperidinium pallidum	0.001	57-62	80	82896	675.9	102.4
Protoperidinium pellucidum	0.001	30-54	42-52	21793	226.3	34.3
Protoperidinium steinii	<0.001	30-42	30-50	15741	173.4	26.3
Scrippsiella CPX	0.039	16-27	16-27	5552	73.8	11.2
Scrippsiella trochoidea	0.022	25-28	25-30	6530	84.3	12.8
Torodinium robustum	<0.001	20	65	6803	87.2	13.2
Apedinella radians	<0.001	8-9	8-9	321	4.1	0.6
Dictyocha fibula	0.002	25-35	25-35	7065	74.1	11.2
Dictyocha speculum	0.006	20-35	20-35	5811	61.6	9.3
Dinobryon divergens	<0.001	4	7	59	0.8	0.1
Dinobryon faculiferum	<0.001	3	7	33	0.5	0.1
Plagioselmis	<0.001	5-7	7-9	104	1.4	0.2
Pseudopedinella sp.	<0.001	8-12	8-12	268	3.4	0.5
Teleaulax spp.	0.001	5-8	11-19	185	2.4	0.4
Merismopedia	<0.001	0.5	3	45	0.6	0.1
Oscillatoriales	<0.001	7	100	3847	41.8	6.3
Pseudanabaena limnetica	<0.001	1.5	100	177	2.3	0.4
Flagellates (spp.)	0.003	3-20	3-20	626	7.6	1.2
Unicell (spp.)	0.001	3-10	3-10	172	2.3	0.3
Laboea strobila	0.054	30-45	60-90	37033	350.9	53.2
Mesodinium rubrum	0.004	20-55	20-55	51441	477.7	72.4

Table S2 Average biovolume ($mm^3\ L^{-1}$), carbon > 3 µm (µM C), percent (%) of total carbon > 3 µm, and percent of total POC (> 0.7 µm), of the dominating groups during August and September light and day incubations. The groups include diatoms (all identified), assigned chain-forming diatoms (*Asterionellopsis glacialis*, *Chaetoceros* spp. > 8 µm, other chain-forming diatoms 5-20 µm), dinoflagellates (all identified), assigned dinoflagellates (*Tripos/Ceratium* spp.), other organisms > 3 µm, and all organisms > 3 µm.

	**August**				**September**			
	Biovolume ($mm^3\ L^{-1}$)	Carbon > 3 µm	Percent of carbon > 3 µm	Percent of total POC	Biovolume ($mm^3\ L^{-1}$)	Carbon > 3 µm	Percent of carbon > 3 µm	Percent of total POC
Diatoms	0.05	0.34	13	2	0.84	4.19	30	11
Assigned chain-forming diatoms	-	-	-	-	0.14	2.23	16	6
Dinoflagellates	0.18	2.04	77	10	0.91	8.93	64	24
Assigned dinoflagellates	-	-	-	-	0.44	3.98	29	11
Other organisms > 3 µm	0.04	0.27	10	1	0.07	0.72	5	2
All organisms > 3 µm	0.27	2.65	100	13	1.82	13.85	100	37

Table S3 Dissolved inorganic nitrate, nitrite, phosphate, silicate, and ammonium concentrations *in situ* (µM). The samples were collected in the morning (light) and evening (dark), during August and September, (average ± SD), $n = 5$.

	Nitrate *in situ*	Nitrite *in situ*	Phosphate *in situ*	Silicate *in situ*	Ammonium *in situ*
August Light	0.59 ± 0.13	0.04 ± 0.01	0.30 ± 0.17	3.91 ± 0.34	0.52 ± 0.27
August Dark	0.49 ± 0.14	0.08 ± 0.00	0.10 ± 0.03	4.82 ± 1.38	0.29 ± 0.07
September Light	0.21 ± 0.04	0.02 ± 0.00	0.07 ± 0.01	5.72 ± 0.32	0.46 ± 0.10
September Dark	0.17 ± 0.03	0.02 ± 0.00	0.04 ± 0.02	3.58 ± 0.34	0.39 ± 0.08

Table S4 Particulate organic carbon (POC), nitrogen (PON), organic matter ratio (POC:PON), and carbon to nitrogen assimilation ratio (mol:mol) in bulk measurements during light, dark, day and overnight incubations in August and September, (average ± SD), *n* = 3.

		Organic matter			Assimilation ratio
		POC	PON	POC:PON	carbon to nitrogen
August	T0 light	20.7±1.4	3.5±0.1	6.1±0.4	-
	T2 light	19.8±0.6	3.3±0.1	6.0±0.2	2.2±0.2
	T5 light	22.8±1.8	3.7±0.3	6.2±0.5	3.4±0.1
	T12 light	24.4±0.7	3.8±0.2	6.4±0.4	9.5±1.4
	T24 light	27.7±3.4	4.2±0.1	6.5±0.7	10.1±0.3
	T0 dark	20.7±0.5	3.2±0.2	6.5±0.2	-
	T2 dark	21.4±0.4	3.3±0.1	6.6±0.0	0.6±0.0
	T5 dark	22.7±1.5	3.7±0.4	6.1±0.3	0.3±0.1
September	T0 light	35.6±2.3	5.6±0.5	6.3±0.1	-
	T2 light	36.7±1.9	5.8±0.3	6.3±0.1	0.8±0.0
	T5 light	38.5±3.6	6.2±0.8	6.3±0.2	1.5±0.3
	T12 light	39.9±1.6	6.1±0.3	6.5±0.0	13.5±0.2
	T24 light	42.4±2.0	6.6±0.3	6.4±0.1	12.8±0.2
	T0 dark	36.0±0.6	5.0±0.2	7.3±0.2	-
	T2 dark	34.9±1.2	5.0±0.3	7.0±0.2	0.0±0.1
	T5 dark	40.4±5.6	5.9±1.3	7.0±0.5	0.1±0.0

Table S5 Environmental conditions. The salinity, time of sunrise, time of sunset, average light *in situ* at midday during the incubations (lux converted to µmol photons s^{-1} m^{-2}), temperature (°C), and sampling depth (m) in August and September. The timings of the carbon, ammonium and nitrate incubations are also included.

	Salinity	Sunrise	Sunset	Light Midday	Temperature	Sampling depth	Incubations ^{15}N-ammonium + ^{13}C-bicarbonate	Incubations ^{15}N-nitrate + ^{13}C-bicarbonate
**August**								
Light/day/overnight	24.3	05:53	08:39	120	18 °C	5 m	07:00-09:00 (2 h), 07:00-12:00 (5 h)	07:00-19:00 (12 h), 07:00-07:00 (24 h)
Dark	24.6	-	-		-	5 m	19:00-21:00 (2 h), 19:00-24:00 (5 h)	-
**September**								
Light/day/overnight	23.5	06:53	07:21	50	16 °C	3 m	07:00-09:00 (2 h), 07:00-12:00 (5 h)	07:00-19:00 (12 h), 07:00-07:00 (24 h)
Dark	24.1	-	-		-	3 m	19:00-21:00 (2 h), 19:00-24:00 (5 h)	-

Bacterial grazing in the *Chaetoceros* phycosphere (budget calculations):

Assumptions:
Chaetoceros cell dimensions: 10 μm wide and 20 μm long
The radius of the phycospheres is 100-fold larger that the diatom cells: $1.8*10^{-5}$ mL
Bacterial abundance in ambient water: $3*10^{6}$ bacteria mL^{-1}*
Bacterial abundance in phycosphere: $3*10^{8}$ bacteria mL^{-1}.
Bacterial size 0.5 μm^3 (similar to attached bacteria)**
Bacterial N-content: 2.7 fmol N $cell^{-1}$ (similar to attached bacteria)**+
Bacterial growth rate in phycopheres :1 d^{-1}
Bacterial N assimilation during growth: 0.12 fmol N cell h^{-1}
Bacterial N content within phycosphere: 3E8 ×2.7 fmol N/cell = 1.5E4 fmol N

Grazing of bacteria by protozoa to produce 200 fmol h^{-1} (50% net release): $2\times200/1.5*10^{4}$= 0.027 h^{-1}
*)Li, 1998
**) Bacterial size and bacterial carbon content as in Grossart et al, 2003 who reported grazing rates in diatom agg: 0.012±0.006 (range: 0.004-0.038 h^{-1}; rates decrease with increasing agg size
+). Assuming a bacterial C:N ratio of 4.5

Gradient of ammonium across boundary layer of single bacterial cells if bacteria release ammonium during ammonification of DON:
Assumptions:
Chaetoceros cell dimensions: 10 μm wide and 20 μm long
The radius of the phycospheres is 100-fold larger that the diatom cells: $1.8*10^{-5}$ mL
$3*10^{6}$ bacteria in the ambient water and 100-fold enrichment in the phycophere.
Bacterial size 0.5 um^3 (similar to attached bacteria)
Bacterial average radius: 0.37 μm
Bacterial N-content: 2.7 fmol N/cell (similar to attached bacteria)
Bacterial growth rate in phycopheres :1 d^{-1}
Bacterial N assimilation during growth: 0.12 fmol N cell h^{-1}
Diffusion coefficient of ammonium: $1.6*10^{-5}$ cm^2 s^{-1}
Gradient of ammonium at (25% net release during ammonification and growth) across the boundary layer of bacterial cell: (0.33× 0.12 fmol N h^{-1}/3600 s/h^{-1})/(4p× $0.37*10^{-4}$ cm×$1.6*10^{-5}$ cm^2 s^{-1}) = 1.4 nM

References:
Li, WKW (1998) Annual average abundance of heterotrophic bacteria and Synechococcus in surface ocean waters. Limnol. Oceanogr. 43: 1746-1756

Grossart H-P, Hietanen S, Ploug H. 2003. Microbial dynamics on natural diatom aggregates in Øresund, Denmark. Mar. Ecol. Prog. Ser. 249: 69-78.
